# Ultrasonic-Assisted Extraction of *Dictyophora rubrovolvata* Volva Proteins: Process Optimization, Structural Characterization, Intermolecular Forces, and Functional Properties

**DOI:** 10.3390/foods13081265

**Published:** 2024-04-20

**Authors:** Yongqing Zhang, Shinan Wei, Qinqin Xiong, Lingshuai Meng, Ying Li, Yonghui Ge, Ming Guo, Heng Luo, Dong Lin

**Affiliations:** 1 State Key Laboratory of Functions and Applications of Medicinal Plants, Guizhou Medical University, Guiyang 550014, China; gyuzhyq@163.com (Y.Z.); 15761632673@163.com (S.W.); 18208490421@163.com (Q.X.); 2 Guizhou Higher Education Key Laboratory of Functional Food, Guizhou Engineering Research Center for Fruit Processing, College of Food Science and Engineering, Guiyang University, Guiyang 550005, China; 15040260380@163.com (L.M.); li.ying.1990@163.com (Y.L.); skyge@163.com (Y.G.); 3 Guizhou Jin Chan Da Shan Biotechnology Company Limited, Bijie 553300, China; guoming@163.com

**Keywords:** *Dictyophora rubrovolvata*, edible mushroom proteins, ultrasonic extraction, functional properties, intermolecular forces

## Abstract

*Dictyophora rubrovolvata* volva, an agricultural by-product, is often directly discarded resulting in environmental pollution and waste of the proteins’ resources. In this study, *D. rubrovolvata* volva proteins (DRVPs) were recovered using the ultrasound-assisted extraction (UAE) method. Based on one-way tests, orthogonal tests were conducted to identify the effects of the material–liquid ratio, pH, extraction time, and ultrasonic power on the extraction rate of DRVPs. Moreover, the impact of UAE on the physicochemical properties, structure characteristics, intermolecular forces, and functional attributes of DRVPs were also examined. The maximum protein extraction rate was achieved at 43.34% under the best extraction conditions of UAE (1:20 g/mL, pH 11, 25 min, and 550 W). UAE significantly altered proteins’ morphology and molecular size compared to the conventional alkaline method. Furthermore, while UAE did not affect the primary structure, it dramatically changed the secondary and tertiary structure of DRVPs. Approximately 13.42% of the compact secondary structures (α-helices and β-sheets) underwent a transition to looser structures (β-turns and random coils), resulting in the exposure of hydrophobic groups previously concealed within the molecule’s core. In addition, the driving forces maintaining and stabilizing the sonicated protein aggregates mainly involved hydrophobic forces, disulfide bonding, and hydrogen bonding interactions. Under specific pH and temperature conditions, the water holding capacity, oil holding capacity, foaming capacity and stability, emulsion activity, and stability of UAE increased significantly from 2.01 g/g to 2.52 g/g, 3.90 g/g to 5.53 g/g, 92.56% to 111.90%, 58.97% to 89.36%, 13.85% to 15.37%, and 100.22% to 136.53%, respectively, compared to conventional alkali extraction. The findings contributed to a new approach for the high-value utilization of agricultural waste from *D. rubrovolvata.*

## 1. Introduction

The next 30 years are expected to witness a significant increase in the demand for high-quality proteins due to the fast expansion of the world’s population and growing consciousness about nutrition. Nowadays, food proteins are mainly derived from animals and plants. However, the high cost of animal breeding, along with greenhouse gas production and environmental pollution, as well as the absence of one or more essential amino acids in plant proteins, have limited their application [1]. Given this, new alternative sources of proteins are being explored. Edible mushrooms are preferred by consumers all over the world owing to their delicious taste and rich nutrition (e.g., proteins, dietary fibers, micronutrients). At present, benefiting from the well-established artificial cultivation technology, the edible fungi industry has been flourishing across the world, and the annual production exceeds 40 million tons merely in China. In comparison with animal raising and crop planting, the cultivation of edible mushrooms is low-cost and eco-friendly with a shorter growth cycle. Edible mushrooms are considered promising food protein sources due to their high protein content (approximately 19–35% in dry weight) and well-balanced ratio of amino acids, effectively meeting the nutritional requirements of essential amino acids for the human body [2].

*Dictyophora rubrovolvata*, a type of saprophytic fungus, is classified as a member of the *Phallus* species [3]. Previous research has indicated that *D. rubrovolvata* is abundant in nutrients such as proteins, polysaccharides, flavonoids, and terpenoids, which possess physiological effects including immunomodulation [4], antioxidant properties [5], anti-tumor effects [6], and antimicrobial properties [7]. Its appealing appearance, delightful taste, and exceptional nutritional content have made *D. rubrovolvata* highly sought after in China, Japan, Korea, and various other Asian nations. Due to its high market demand and significant commercial worth, *D. rubrovolvata* has emerged as a prominent and thriving species of edible fungus in the southwestern provinces of China, namely Guizhou, Yunnan, and Sichuan. In Guizhou province alone, the annual production reaches approximately 10,000 tons. About 35% of its overall weight is made up of the stipe and the veil, which are the edible parts [2]. However, the volva of *D. rubrovolvata*, which makes up approximately 40% of the entire fruiting body, is discarded after harvesting, leading to significant environmental pollution and wastage of bio-resources [3]. Zhuang and Sun [2] revealed that the crude proteins content in *D. rubrovolvata* volva was 26.74%, implicating its potential as a source of high-quality protein.

The use of ultrasound-assisted extraction (UAE) has become prevalent in protein recovery due to its benefits, including efficient extraction, time and solvent conservation, ease of operation, and environmental friendliness [8]. Ultrasonic waves propagate through the fluid medium as pressure oscillations, generating numerous rapidly expanding cavitation cavities. The cavitation region experiences transient high temperatures (5000 k), high pressures (1000 atm), shock waves, turbulence, and shear due to the expansion and rupture of cavitation bubbles [8]. On one hand, cavitation causes the bio-matrix to perforate and fragment, enhancing solvent accessibility and improving protein extraction efficiency [9]. On the other hand, the cavitation effect partially unfolds the proteins and alters their advanced structure (secondary, tertiary, and quaternary structures), disrupting the non-covalent interactions among proteins molecules [10]. These ultrasonic structural modifications can greatly enhance the functional properties of proteins for various processing purposes, including solubility, foaming, emulsification, and gelation. Reports indicated that sonication is effective in modifying the properties of proteins in soy [10], cod [11], and peanuts [12]. Therefore, it is hypothesized that UAE applied to *D. rubrovolvata* volva might substantially improve its structural and functional properties.

Currently, there are few studies about the effects of UAE on *D. rubrovolvata* volva proteins (DRVPs). Therefore, the purpose of this work was to study the DRVP modification using UAE in terms of physicochemical and structural characterization, intermolecular forces, and functional properties. The results will establish the groundwork for the advancement of DRVPs as a novel food protein source and its utilization in the food sector. It could provide a significant reference for realizing the sustainable development goal of the *D. rubrovolvata* industry.

## 2. Materials and Methods

### 2.1. Materials

*D. rubrovolvata* volva was kindly provided by Guizhou Jin Chan Da Shan Biotechnology Co., Ltd. (Bijie, China). Tris (hydroxymethyl) aminomethane, sodium dodecyl sulfate (SDS), β-mercaptoethanol (β-ME), and bromophenol blue were purchased from Sangon Bioengineering Co., Ltd. (Shanghai, China). Macklin Biochemical Technology Co., Ltd. (Shanghai, China) provided 5,5′-Dithio bis-(2-nitrobenzoic acid) (DTNB) and 8-Anilino-1-naphthalenesulfonic acid (ANS). All other analytical-grade chemicals and reagents, unless otherwise specified, were obtained from Sinopharm Chemical Reagent Co., Ltd. (Shanghai, China).

### 2.2. Optimization of UAE Process of DPVP

#### 2.2.1. Extraction Method and Determination of Proteins Extraction Rate

Fresh *D. rubrovolvata* volvas were washed, freeze-dried, pulverized, sieved (60 mesh), and then degreased with petroleum ether (1:2, *w*/*v*) for 24 h. We weighed accurately 10.00 g of defatted powder (proteins content m_1_, determined using the Kjeldahl method) into a beaker and mixed it with a specific volume of distilled water. Afterwards, the mixtures were adjusted to the assigned pH with 1 M HCl or NaOH. The extraction was carried out for a certain period at a specified power in a water bath ultrasonic reactor (KQ 3200DB, 40 kHz, Kunshan Ultrasonic Instrument Co., Ltd., Kunshan, China). Upon completion of the extraction, the supernatant (proteins content m_2_, determined using the Coomassie brilliant blue method) was collected using centrifugation at 5000 r/min for 20 min. After the pH adjustment to the proteins isoelectric point (pH 2.5) with 1 M HCl, the sediment was collected using centrifugation again (5000 r/min, 20 min). Then, the precipitates were lyophilized in a vacuum freeze dryer (LC-12N-50A, Lichen Instrument Technology Co., Ltd., Shaoxing, China) for 24 h and stored in a refrigerator at −20 °C for further analysis. The protein extraction rate was calculated using the following equation: protein extraction rate (%) = m_2_/m_1_ × 100 [13].

#### 2.2.2. Single-Factor and Orthogonal Experiments Design

The effects of four factors (pH, ultrasonic time, ultrasonic power, and material–liquid ratio) on the extraction rate of DRVPs were investigated in the one-factor experiments, respectively [14]. The reference conditions were set at pH 11, ultrasound power 550 W, ultrasound time 25 min, and material–liquid ratio 1:10. Only one factor was varied in each trial, and the other factor levels were kept constant. The ranges of levels for each factor are as follows: pH (9.0, 9.5, 10.0, 10.5, 11.0, 11.5), ultrasound time (10, 15, 20, 25, 30 min), ultrasound power (330, 385, 440, 495, 550 W), and material–liquid ratio (1:10, 1:15, 1:20, 1:25, 1:30). Based on the results of the one-factor test, a four-factor three-level orthogonal test was conducted with protein extraction rate as the response variable. The factors and levels of the orthogonal test were listed in Table S1. The optimal UAE process of DRVPs was obtained and validated by integrating it with the range analysis.

### 2.3. Determinations of the Physicochemical Properties

#### 2.3.1. Microstructures

The scanning electron microscope (SEM) (Gemini500, Carl Zeiss AG, Oberkochen, Germany), magnified at 30,000 times, was used to observe the microstructures of the samples. After applying conductive adhesive, the lyophilized proteins powders were attached to the sample stage. Subsequently, the sample micromorphology was observed and photographed at an accelerating voltage of 15 kV following the gold sputtering process [15].

#### 2.3.2. Turbidity

The samples were prepared as a 5 mg/mL solution in water, and their absorbance at 660 nm was measured at room temperature using a UV–Vis spectrophotometer (Cary 60, Agilent Technologies, Santa Clara, CA, USA) [16].

#### 2.3.3. Particle Size Distribution and Zeta Potential

Minor modifications were made to Zhong’s and Xiong’s [16] method for analyzing particle size and zeta potential. The 1 mg/mL of proteins was dissolved in phosphate-buffered saline (0.01 M, pH 7.0). At 25 °C, the Malvern Company’s laser particle size analyzer (Nano ZS90, London, UK) was employed to ascertain the distribution of particle sizes and zeta potential.

#### 2.3.4. Thermal Properties

The thermal characteristics of the proteins samples were assessed employing a DSC4000 differential scanning calorimeter (PerkinElmer Ltd., Waltham, MA, USA). The approach described by Wang et al. [9] was followed. Nitrogen was passed through at a flow rate of 40 mL/min, and an aluminum pan was used to enclose 5 mg of the sample. Using an aluminum pan devoid of any contents as a control, the temperature was gradually increased at a speed of 5 °C/min across the temperature range of 20 to 180 °C. The thermal profiles were scanned and graphed during this process.

### 2.4. Characterization of the Structures

#### 2.4.1. Amino Acid Profile

The samples were analyzed for their amino acid makeup following the procedure outlined by Eze, Chatzifragkou, and Charalampopoulos [17]. More specifically, 80 mg of proteins powder was combined with HCl (6 M, 10 mL) in a hydrolysis tube and subjected to hydrolysis at 110 °C for 24 h under a nitrogen atmosphere. After passing through a 0.45 µm membrane, the hydrolysis products underwent filtration. Subsequently, amino acid content was monitored with an automated amino acid analyzer (L-8900, Hitachi Co., Tokyo, Japan).

#### 2.4.2. Molecular Weight Distribution

The sodium dodecyl sulfate–polyacrylamide gel electrophoresis (SDS-PAGE) was employed to visualize the molecular weight distribution of DRVPs. The SDS-PAGE was carried out following the procedure described by Zhong and Xiong [16]. There were two types of gels used, 12% separating gel and 5% concentrating gel. The proteins solution, with a concentration of 5 mg/mL, was mixed with buffer containing 0.05 M Tris-HCl buffer, 5% glycerol, 1% SDS, 2.5% β-ME, and 0.02% bromophenol blue in an equal volume. The mixture was then heated at 95 °C for 5 min to denature. The sample (20 μL) was placed into the gel and the electrophoresis was conducted at a steady voltage of 100 V. The gel was dyed using BeyoBlue Ultrafast Staining Solution (P0017F, Beyotime, Nantong, China) while being shaken for 1 h, then washed in distilled water for 2 h to remove the color. To determine the molecular weight of the proteins samples, a non-prestained protein marker (P0060M, Beyotime, Nantong, China) with a range of 10 to 150 kDa was utilized.

#### 2.4.3. Secondary Structure

The fourier transform infrared (FTIR) spectrometer (Spectrum Two, Perkin Elmer Ltd., Waltham, MA, USA) was utilized to measure proteins samples, scanning 32 times at a resolution of 4 cm^−1^ within a wavelength range of 500 to 4000 cm^−1^. The absorption peaks in the amide I band (1600–1700 cm^−1^) were subjected to Gaussian deconvolution using Peakfit software (v4.12, Thermo Electron Co., Waltham, MA, USA). The area of each subpeak was used to calculate the percentage of each secondary structure [18].

#### 2.4.4. Surface Hydrophobicity (H_0_)

A hydrophobic fluorescent probe created using the ANS was employed to determine the surface hydrophobicity of the proteins sample, following the method described by Li et al. [19]. In short, proteins dispersion (1 mg/mL) was produced using phosphate buffer (0.01 M, pH 7.0). Then, 4 mL proteins solution was mixed with 20 µL freshly prepared ANS solution (8.0 M). The blend underwent a reaction for 20 min at ambient temperature while being shielded from light. The fluorescence intensity in the emission wavelength range of 470–520 nm was recorded with 370 nm as the excitation wavelength using a fluorescence spectrophotometer (F-320, Tianjin Gangdong Technology Co., Tianjin, China).

#### 2.4.5. Free Sulfhydryl (SH) Content

With slight modifications, Ellman’s reagent was used to determine the SH content [19]. The Tris-glycine buffer (pH 8.0) was configured as follows: 0.086 M Tris, 0.09 M glycine, 4 mM EDTA-Na2, 0.5% SDS and 8 M urea. Ellman’s reagent was made by combining 20 mg of DTNB with 5 mL of the buffer above. To 4 mL of proteins solution (2 mg/mL), Ellman’s reagent was added in the amount of 40 µL. Following vortex, the reaction was conducted at 25 °C for 15 min in a light-free environment. The absorbance value at 412 nm was recorded using a Cary 60 UV spectrophotometer (Agilent Technologies, Santa Clara, CA, USA). The precise SH content was determined using the following calculation.

(1) $$\mathrm{SH}\;\mathrm{content}\;(\mu \mathrm{mol}/g)=\frac{73.53\times A_{412}\times D}{C}$$

where A_412_ represents the absorbance value of the sample at 412 nm, D represents the sample dilution factor, and C represents the mass concentration of the proteins (mg/mL).

### 2.5. Proteins Aggregates Dissociation Test

The method was modified slightly from Wang et al. [20]. Specifically, the proteins solution of 2 mg/mL was combined with various concentrations of bond dissociation agents (HCl/NaOH, SDS, urea, and β-ME) at a volume ratio of 1:9 and allowed to stand overnight. The size distribution of the sample was then recorded with a laser nanoparticle sizer (Nano ZS90, Malvern Company, Malvern, UK). A proteins dispersion without bond dissociation reagents was used as a control.

### 2.6. Measurements of Functional Properties

#### 2.6.1. Solubility

Solubility was determined according to Alavi et al. [21] with slight modifications. The proteins samples were 1 mg/mL in distilled water and then pH values of 2, 4, 6, 8, and 10 were adjusted. To collect the supernatant, the mixture was centrifuged at 5000 r/min for 20 min following 10 min vortex. The Coomassie Brilliant Blue method was employed to determine the proteins amount in the supernatant. The protein solubility was calculated using the provided Equation (2):

(2) $$\mathrm{Protein}\;\mathrm{solubility}\;(\%)=\frac{C_{1}}{C_{0}}\times 100$$

where C_1_ is the proteins. concentration in the supernatant (mg/mL), and C_0_ is the total proteins concentration (mg/mL).

#### 2.6.2. Water Holding Capacity (WHC) and Oil Holding Capacity (OHC) 

After making slight adjustments, the evaluation of WHC and OHC was conducted using Huang et al.’s [15] method. Mix 10 mL of distilled water or soybean oil in a centrifuge tube containing 1 g of proteins sample. Then, the tubes were placed in the water baths at various temperatures (30, 40, 50, 60, 70 °C) for 30 min. Subsequently, they were centrifuged at a speed of 5000 r/min for 20 min, and the resulting supernatant was removed. WHC and OHC were calculated using Equation (3):

(3) $$\mathrm{WHC}/\mathrm{OHC}\;(g/g)=\frac{W_{3}-W_{2}}{W_{1}}$$

where W_3_ is the weight of the empty tube plus the absorbed water/oil proteins sample (g), W_2_ is the weight of the empty tube plus the proteins sample (g), and W_1_ is the weight of the proteins sample (g).

#### 2.6.3. Emulsion Activity Index (EAI) and Emulsion Stability Index (ESI)

EAI and ESI were assessed as described by Li et al. [19] with minor modifications. To create an emulsion, 2 mL of soybean oil was added to 8 mL of proteins dispersion. The pH of the emulsion was then modified to various levels (2, 4, 6, 8, and 10), and homogenization was performed for 2 min at 10,000 r/min using a homogenizer from IKA, Germany (T25 digital ULTRA-TURRAX). A 50 μL aliquot was aspirated from the lower part of the tube (0 and 10 min) and diluted 100 times with SDS solution (*w*/*v*, 0.1%). Afterward, it was mixed by vortexing for 10 s. A spectrophotometer was used to measure the absorbance of the diluted emulsion at 500 nm. The calculation of EAI and ESI was performed using Equations (4) and (5), respectively:

(4) $$\mathrm{EAI}\;(m^{2}/g)=\frac{2\times 2.303\times N{\times A}_{0}}{C\times L\times \varphi \times 10000}$$

(5) $$\mathrm{ESI}\;(\min)=\frac{A_{0}}{A_{0}-A_{10}}\times 10$$

where N represents the dilution coefficient (100), C is the proteins concentration (g/mL), L represents the optical path (1 cm), φ represents a fraction of the oil phase (0.2), and A_0_, A_10_ denote absorbance at 0 and 10 min.

#### 2.6.4. Foaming Capacity (FC) and Foaming Stability (FS)

FC and FS were determined according to the method outlined by Li et al. [22]. After making slight adjustments, DRVP solutions (14 mL, 5 mg/mL) with varying pH levels (2, 4, 6, 8, and 10) were agitated for 2 min at a speed of 10,000 r/min using a T25 digital homogenizer (ULTRA-TURRAX, IKA, Staufen, Germany). Subsequently, the foam volume (V_0_) was promptly measured using a graduated cylinder. After 30 min of settling, the foam volume (V_30_) was checked again. The equations below were used to calculate FC and FS.

(6) $$\mathrm{FC}(\%)=\frac{V_{1}}{V_{0}}\times 100$$

(7) $$\mathrm{FS}(\%)=\frac{V_{30}}{V_{1}}\times 100$$

where V_0_ is the volume (mL) before homogenization, V_1_ is the volume (mL) after homogenization, and V_30_ is the volume (mL) after 30 min of standing.

### 2.7. Statistical Analysis

The trials were performed thrice, and the outcomes were displayed as the means ± standard deviation. Origin 2018 software from Origin Lab Inc. (Northampton, MA, USA) was utilized to arrange and graph the data. Statistical analysis was conducted using SPSS software (version 18.0, SPSS Inc., Chicago, IL, USA). The means of two groups were analyzed using the Student’s *t*-test. For multiple comparisons, one-way analysis of variance (ANOVA) followed by Duncan’s multiple range test was performed. The results were deemed significantly distinct with a *p*-value less than 0.05.

## 3. Results and Discussion

### 3.1. Optimization of the Extraction Process of DRVPs

**Figure 1:**
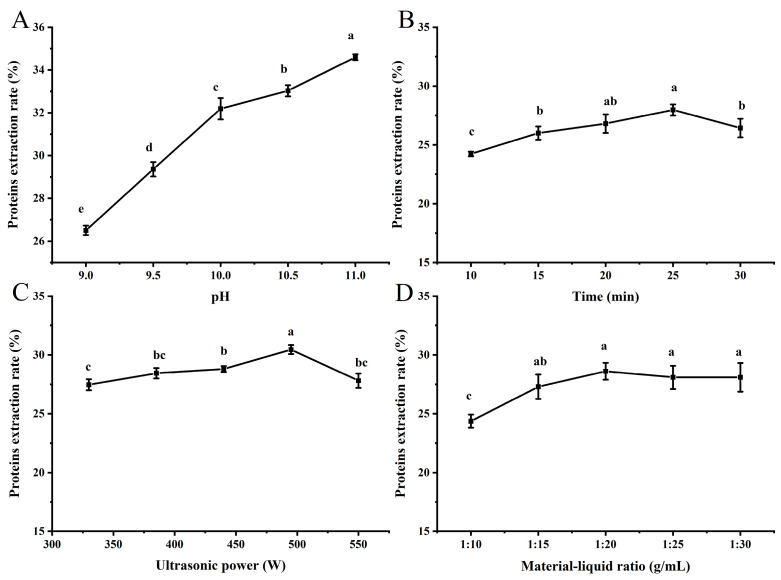
Effects of different process conditions on protein extraction rate of DRVPs. (**A**) pH, (**B**) time, (**C**) ultrasonic power, and (**D**) material–liquid ratio. Among different levels of the same single factor, the different letters indicate a significant difference (*p* < 0.05).

Different extraction conditions have varying effects on the extractability of proteins. To improve the efficiency of proteins preparation, it was evaluated how pH, time (min), ultrasonic power (W), and material–liquid ratio (g/mL) affected the DRVP extraction rate. As shown in Figure 1A, the protein extraction rate increased gradually with the rise in pH. Proteins are negatively charged in an alkaline environment and repel each other, which helps them to increase their solubility [14]. The highest protein extraction rate of 34.60% was achieved at pH = 11. With the prolongation of ultrasonication time, the protein extraction rate was first increased and then decreased. The highest protein extraction rate of 27.97% was reached at 25 min (Figure 1B). The shock wave and shear force arising from ultrasonic cavitation broke the cell wall and promoted the release of proteins. However, too-long ultrasonication time can cause protein aggregation and precipitation, thereby resulting in a decline in the protein extraction rate [23]. With the elevation of power, the cavitation effect also increased, significantly enhancing the mass transfer efficiency. Consequently, as the ultrasonic power rose, the protein extraction rate increased accordingly. At 495 W, the extraction rate reached the maximum value of 30.45% (Figure 1C). However, when the ultrasonic power was increased further, the extraction rate decreased instead (Figure 1C). This is because the local thermal effect caused by high-intensity cavitation can lead to protein degradation, thus reducing the extraction rate [13]. Figure 1D describes the effect of the material–liquid ratio on protein extraction rate. With the increase in solvent amount, the powders were in fuller contact with the solvent, which favored the protein extraction. Up to a material–liquid ratio of 1:20, a maximum value of 28.61% was reached. Continuing to increase the solvent volume, the protein extraction rate no longer increased, owing to the progressive dilution of the ultrasound energy density.

Concerning the results of the single-factor tests, the L_9_(3^4^) orthogonal table was designed to optimize the extraction process of DRVPs. The experimental design and results are listed in Table S2. Based on the relative magnitude of the R value and the sum of squares, the order of the factors affecting the protein extraction rate was obtained as follows: pH > ultrasonic power > material–liquid ratio > ultrasonic time (Tables S2 and S3). The variance analysis revealed that pH (*p* < 0.01) and ultrasonic power (*p* < 0.01) had a significant effect on protein yield, while ultrasonic time (*p* > 0.05) and material-liquid ratio (*p* > 0.05) did not show any significant effect (Table S3). This suggested that the increase in protein extraction rate was mainly dependent on pH and ultrasonic power. A prolonged sonication time and an increase in the liquid-liquid ratio had a limited effect on the extractability. According to the k-value analysis, the optimization condition for the extraction of DRVP was A_3_B_2_C_3_D_2_ (Table S2), namely: pH = 11, ultrasonic power of 550 W, ultrasonic time of 25 min, and material-liquid ratio of 1:20, which resulted in the highest protein extraction rate of 43.37%. Proteins extracted in the above condition (designated as U-DRVP) were used for subsequent structural characterization and functional property evaluation, while those extracted by conventional alkaline extraction without UAE (designated as C-DRVP, 32.80% protein extraction rate) were employed as control.

### 3.2. Physical and Chemical Properties of U-DRVP

#### 3.2.1. SEM Analysis

**Figure 2:**
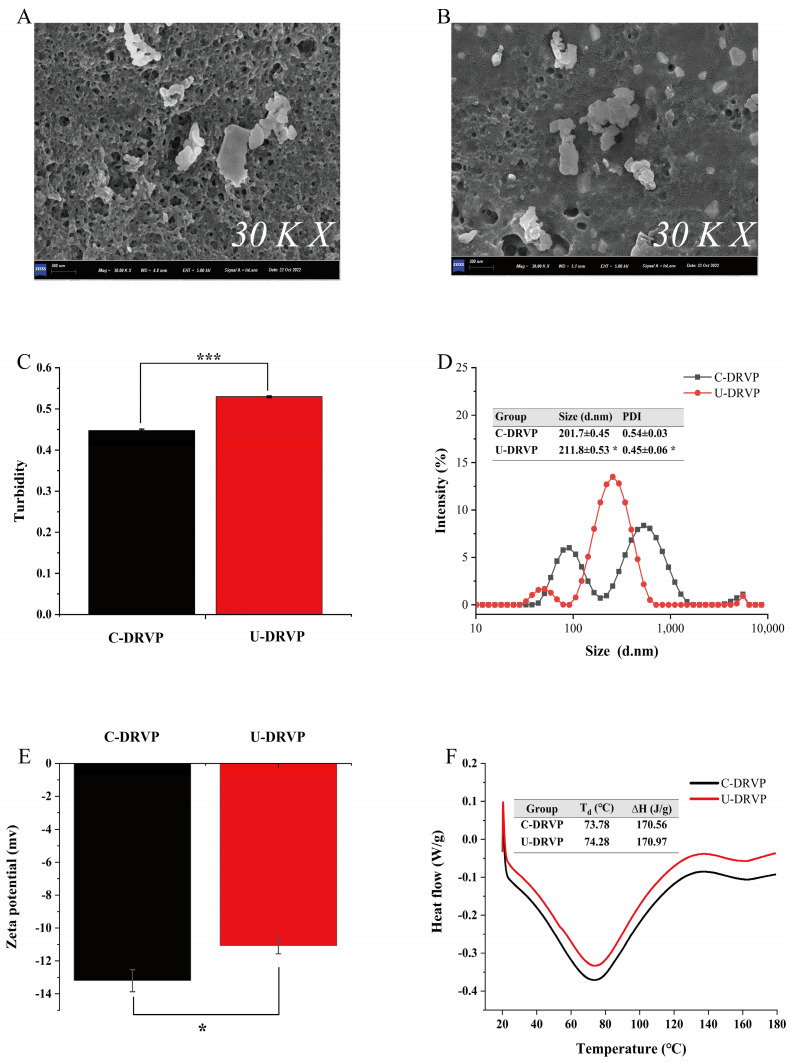
Physicochemical properties of C-DRVP and U-DRVP. (**A**,**B**) SEM images, (**C**) turbidity, (**D**) particle size distribution, (**E**) zeta potential, and (**F**) DSC. Note: The statistical method is Student’s *t*-test. The symbols indicate statistical significance between the means of two groups: *: *p* < 0.05, ***: *p* < 0.001. C-DRVP, *D. rubrovolvata* volva proteins extracted using conventional alkaline extraction without UAE; U-DRVP, *D. rubrovolvata* volva proteins extracted using UAE.

The changes in the micromorphology of the proteins surface after UAE were examined with SEM. As shown in Figure 2A,B, UAE noticeably altered the apparent morphology of DRVP. C-DRVP showed a highly cross-linked, porous, and rough surface (Figure 2A), whereas the surface of U-DRVP was more dense, smooth, and flat with better continuity (Figure 2B). On the one hand, ultrasonic cavitation induced changes in the structure of DRVP, exposing the functional groups buried inside the molecules and destroying the original aggregation pattern between proteins molecules [24]. On the other hand, the external energy afforded by ultrasound increased the collision frequency between proteins molecules [25], approaching each other via new molecular interactions (non-covalent or/and covalent interactions) [15], accomplishing the reconfiguration of the protein aggregates, and eventually presenting the microstructure shown in Figure 2B. Lv et al. [26] also reported similar findings.

#### 3.2.2. Turbidity, Particle Size Distribution, and Zeta-Potential Analysis

Larger-sized colloidal particles usually have a greater light scattering capacity and proportionally higher turbidity values [16]. As shown in Figure 2C, the turbidity values for C-DRVP and U-DRVP were 0.45 and 0.53, respectively. Higher turbidity values for U-DRVP suggested larger molecular sizes. Consistent with the turbidity measurements, the average particle size of U-DRVP was significantly elevated from 201.7 nm to 211.8 nm compared to that of C-DRVP (Figure 2D). The particle size distribution of U-DRVP appeared as a single peak, while C-DRVP as a double peak. Correspondingly, the PDI values were 0.45 and 0.54, respectively, indicating that U-DRVP had a more homogeneous particle size distribution. The results above can be attributed to two points: for one thing, the ultrasound-induced shear and microjet loosened the proteins structure, exposing internal active groups that favor molecular aggregation [27]; for another, the thermal effect produced with ultrasound accelerated the collision and aggregation rate of DRVP particles, which drove them to form more homogeneous and larger aggregates by self-assembly [16].

The zeta potential could reflect the charged properties of the particle surface [27]. As illustrated in Figure 2E, the potential of U-DRVP rose from −13.20 mV to −11.07 mV in comparison with C-DRVP. In general, the higher potential of negatively charged particles implies less intermolecular repulsion and allows for easier formation of larger particles, which is confirmed by the results of turbidity and average particle size (Figure 2C,D). However, the opposite finding was observed by Huang et al. [15], who claimed that sonication caused the fragmentation of the proteins into smaller pieces, exposing more negatively charged groups inside the molecule, consequently resulting in lower potential values. The contradictory results mentioned above may be because there were differences in proteins sources, power, and duration of sonication in each study.

#### 3.2.3. Differential Scanning Calorimetry (DSC) Analysis

DSC is widely deployed to analyze the thermal behavior of proteins [9]. As depicted in Figure 2F, the denaturation temperature (T_d_) of U-DRVP was slightly elevated compared with C-DRVP, moving from 73.78 °C to 74.28 °C. The denaturing enthalpy change (∆H) followed a similar trend, rising from 170.56 J/g to 170.97 J/g. T_d_ usually reflects the thermal stability of the polymer, and ∆H indicates the overall structural order. Ultrasonic cavitation enhanced intermolecular forces and promoted the reorganization of protein particles into more homogeneous aggregates (Figure 2D), thus requiring higher T_d_ and ∆H to induce protein thermal denaturation [9].

### 3.3. Changes in the Structure of U-DRVPs

#### 3.3.1. Primary Structure 

The nutritional value and functional properties of proteins greatly depend on the composition of amino acids [17]. Hence, the amino acid profiles of U-DRVPs were measured. As indicated in Table 1, U-DRVPs were abundant in aspartic acid, glutamic acid, leucine, threonine, and phenylalanine, leaving cysteine as the lowest ingredient. The proportion of negatively charged amino acids (23.89%) was noticeably higher than that of positively charged amino acids (10.09%), partly accounting for the negative potential of U-DRVPs (Figure 2E). Furthermore, U-DRVPs have great nutritional value due to their richness in essential amino acids (316.53 mg/g, 42.09%), surpassing the percentage recommended by WHO. The high percentage of hydrophobic amino acids (356.41 mg/g, 49.05%) was important for U-DRVPs to exhibit excellent processing functional properties. Compared to C-DRVPs, UAE significantly improved the absolute levels of total amino acids of DRVPs, increasing from 683.90 mg/g to 751.89 mg/g. However, UAE had little change in the proportion of amino acid composition, in agreement with the analyses for okara proteins by Eze, Chatzifragkou, and Charalampopoulos [17].

**Figure 3:**
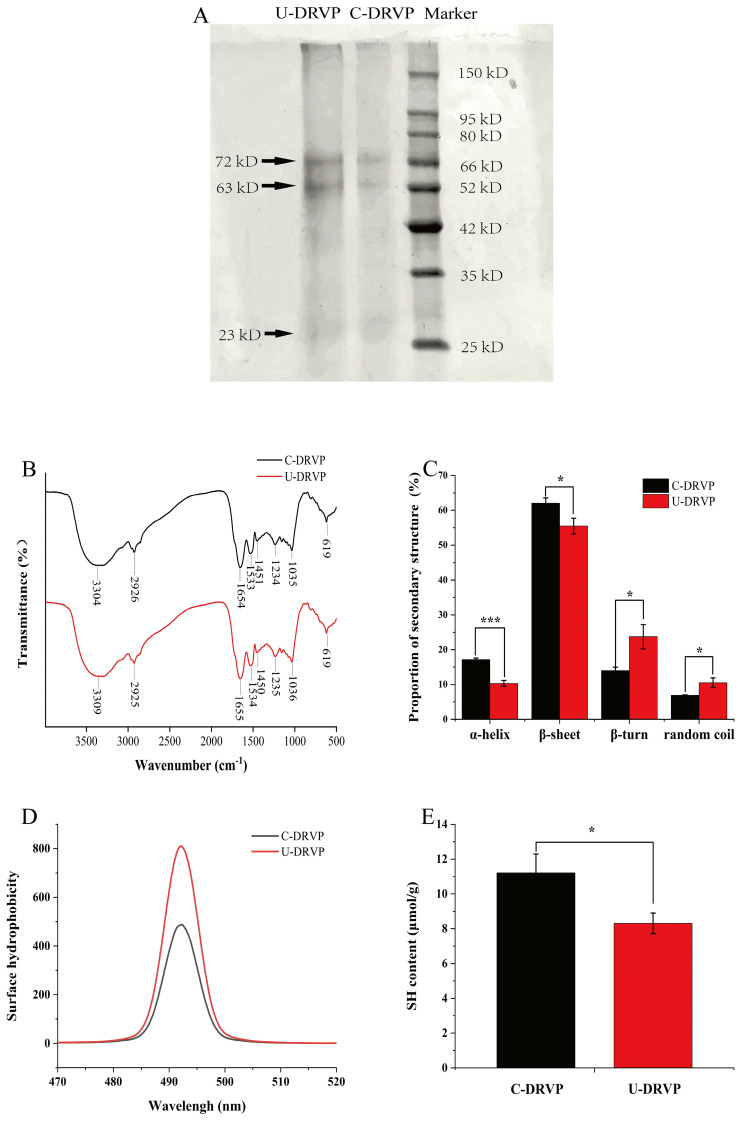
Structural characterization of C-DRVPs and U-DRVPs. (**A**) SDS-PAGE pattern, (**B**) FTIR spectra, (**C**) proportion of secondary structures, (**D**) surface hydrophobicity, (**E**) SH content. Note: The statistical method is Student’s *t*-test. The symbols indicate statistical significance between the means of two groups: *: *p* < 0.05, ***: *p* < 0.001. C-DRVPs, *D. rubrovolvata* volva proteins extracted using conventional alkaline extraction without UAE; U-DRVPs, *D. rubrovolvata* volva proteins extracted using UAE.

The SDS-PAGE was employed to visualize the molecular weight distribution of DRVPs [16]. As indicated in Figure 3A, the protein bands displayed identical patterns regardless of the extraction method. Both protein samples consisted of three major subunit bands with molecular weights located at 23, 63, and 72 kDa, respectively. The ultrasound energy was not strong enough to cleave the peptide bonds, yielding protein fragments with lower molecular weights. Consequently, the primary structure of U-DRVPs remained unchanged. Previous studies also found that the electrophoresis profiles of sonicated amaranth [27] and pea [28] proteins remained unmodified.

#### 3.3.2. Secondary Structure 

FTIR has been commonly used for the detection of characteristic functional groups, which can be applied to quantitatively measure the proportions of secondary structure in proteins [18]. Generally, the amide bond (peptide bond) in the proteins backbone exhibits five characteristic absorption bands in FTIR: amide A, amide B, amide I, amide II, and amide III. Upon UAE, the IR absorption at 3304 cm^−1^ (amide A) was blueshifted to 3309 cm^−1^ (Figure 3B). It is inferred that the inter- or intramolecular hydrogen bonding rearrangements induced by sonocavitation affected the N-H stretching vibration. Furthermore, after UAE, the IR absorptions at 1654 (amide I), 1533 (amide II), and 1234 cm^−1^ (amide III) were blue-shifted to 1655, 1534, and 1235 cm^−1^, respectively, suggesting that the stretching and bending vibration profiles of the C=O, C-N, and N-H bonds changed. Anyway, changes in the vibration of the functional group will affect the intramolecular hydrogen bonding and thus modify the percentage of secondary structures [9].

Among the characteristic absorptions of the five amides, the amide I band is the most responsive to alterations in the secondary structure of proteins. Hence, it was chosen for secondary structure fitting analysis [18]. As noted in Figure 3C, β-sheet was the dominant structure in four secondary structures for both proteins. In comparison to C-DRVPs, α-helix and β-sheet were reduced by 6.85% and 6.56%, corresponding to an increase in β-turn and random coil by 9.70% and 3.61% in U-DRVPs. The α-helix and β-sheet were more compact than the β-turn and random coil. As a result, the structure of U-DRVPs was made looser and more flexible, which was significant for it to realize desirable techno-functional properties. The above outcomes may be because the shock waves, turbulence, microjets, and shear generated with ultrasound disrupt the structure of a DRVP, exposing the groups within the molecule, remodeling the hydrogen bonding, and ultimately altering the ratio of the secondary structure [27].

#### 3.3.3. Tertiary Structure

H_0_ can indirectly reflect the degree of tertiary structure modification and denaturation by measuring the amount of hydrophobic clusters exposed to the proteins surface [19]. As revealed in Figure 3D, U-DRVPs had higher ANS fluorescence intensity (H_0_) than C-DRVPs. It is because the ultrasonic cavitation broke down the interaction forces within DRVP, making the structure became more relaxed and stretched. The hydrophobic groups originally situated within the molecule relocated to the surface. The more fully the hydrophobic groups were exposed, then the more ANS probes were bound, and hence higher fluorescence values were observed [29]. Similar results regarding H_0_ were also reported by Li et al. [19].

The variation in SH content reflects dynamic rearrangements in the spatial conformation of proteins [16]. Similarly to H_0_, SH content also impacts proteins’ functional properties, including solubility and emulsification. Thus, the SH content of DRVP was assayed in Figure 3E. Compared to C-DRVPs, the content of SH in U-DRVPs dropped by 2.91 μmol/g. Kang et al. [30] identified that the SH content of chickpea proteins initially rose and then fell with the increase in sonication time, and that the sonication time had a pronounced effect on the SH content. Accordingly, it was speculated that the internal SH was gradually transferred to the surface of DRVPs at the beginning of UAE, causing an increase in SH content. Instead, SH content continued to decrease rapidly after a critical time point. This may be because the energy delivered by ultrasonic cavitation can split water molecules into hydrogen atoms and high-reactivity hydroxyl radicals. Upon oxidation by hydroxyl radicals, cross-linking between DRVP occurred by exchanging thiols for disulfides. This resulted in the formation of soluble protein aggregates (Figure 2C), thereby decreasing the amount of SH [22].

### 3.4. Intermolecular Forces Analysis

Compared with C-DRVPs, UAE was accompanied by a pronounced expansion of U-DRVP aggregates (Figure 2D), which suggested their different aggregation modes among the proteins particles. To further understand the intermolecular forces involved in the formation and maintenance of DRVP aggregates, the influence of different dissociating agents (HCl/NaOH, SDS, urea, and β-ME) on the particle size of the aggregates were determined (Figures S1 and S2).

#### 3.4.1. Electrostatic Interactions

**Figure 4:**
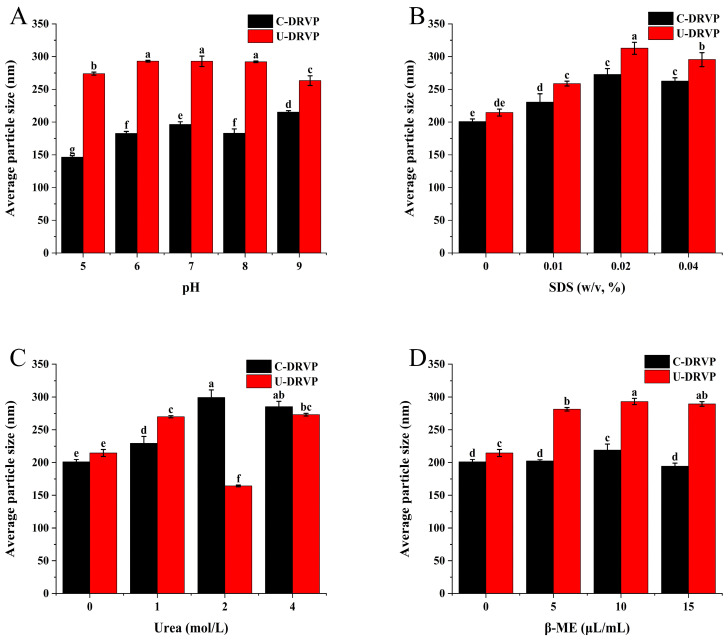
Effects of different dissociation agents on average particle size of C-DRVPs and U-DRVPs. (**A**) pH, (**B**) SDS, (**C**) urea, and (**D**) β-ME. Note: Different letters indicate a significant difference between the means of any two groups (*p* < 0.05). C-DRVPs, *D. rubrovolvata* volva proteins extracted using conventional alkaline extraction without UAE; U-DRVPs, *D. rubrovolvata* volva proteins extracted using UAE.

Modifying pH can change the system charge and subsequently affect the electrostatic interactions between protein particles [31]. Therefore, the role of ionic bonding in DRVP aggregates can be ascertained by varying the pH value. The average particle size of U-DRVPs was slightly reduced in both acidic (pH = 5) and basic (pH = 9) conditions compared to the neutral condition (pH = 7) as illustrated in Figure 4A, whereas in the case of C-DRVPs, the average particle size was reduced under acidic (pH = 5) conditions and slightly increased under alkaline (pH = 9) conditions. These results revealed that the stability of U-DRVP aggregates was affected less by electrostatic interactions (ionic bonding) than that of C-DRVP.

#### 3.4.2. Hydrophobic Interaction

As a surfactant, SDS can interact with nonpolar groups in protein side chains, disrupting hydrophobic forces and causing protein denaturation [20]. Therefore, the impact of SDS on particle size was evaluated to explore the contribution of hydrophobic interaction in maintaining DRVP stability, as depicted in Figure 4B. The results signified that the aggregation of U-DRVPs was related to the concentration of SDS. To be specific, concentration raised from 10 mg/mL to 40 mg/mL, resulting in an increment in average particle size, forming larger aggregates. The hydrophobic forces between U-DRVPs were disrupted by raising the SDS concentration, exposing the hydrophobic regions hidden inside. Subsequently, the long-chain hydrophobic tail of SDS is bound to the exposed region, and the negatively charged head is bound to another positively charged protein via ionic bonding, ultimately forming larger aggregates [20]. A similar effect was also observed in C-DRVP (Figure 4B). Overall, hydrophobic interaction plays an important role in aggregate formation, regardless of sonication.

#### 3.4.3. Hydrogen Bonding 

Urea binds competitively to the amide groups in proteins, forming new hydrogen bonds and disrupting the pre-existing hydrogen bonds in the aggregates [20]. Therefore, urea was added to the aggregates dispersion to probe the role of hydrogen bonding in maintaining DRVP conformation. As seen in Figure 4C, the average particle size of the U-DRVP aggregates followed an increasing–decreasing–increasing trend with urea concentration from 1 M to 4 M. Intramolecular hydrogen bonds were separated by 1 M urea, resulting in swelling of the molecules; 2 M urea disrupted the hydrogen bonds, fragmenting the protein aggregates into smaller particulate; and when the concentration reached 4 M, the urea facilitated intermolecular hydrophobic forces by depriving surfaces moisture, ultimately leading to the formation of larger aggregates [20]. Regarding C-DRVP, its particle size gradually enlarged with increasing urea concentration (Figure 4C). In conclusion, the results confirmed the involvement of hydrogen bonding in the assembly of DRVP aggregates.

#### 3.4.4. Disulfide Bonding

β-ME cleaves disulfide bonds in proteins, releasing more free sulfhydryl groups [20]. Thus, the involvement of disulfide bonds can be examined by adding β-ME. As shown in Figure 4D, simply 5 μL/mL β-ME dramatically raised the particle size of U-DRVP aggregates. This may be due to two reasons: On the one hand, β-ME cleaves disulfide bonds and releases a variety of reactive sites facilitating the action of other intermolecular forces and thus particle size enlargement; on the other hand, β-ME, as a linker, promotes the connection between U-DRVPs containing reactive functional groups [20]. Interestingly, β-ME had a negligible effect on C-DRVP size. The disulfide bonds were essential for U-DRVP aggregates formation, but not for C-DRVPs, which was supported by the data in Figure 4D. Collectively, the forces that maintain the two DRVP aggregates were different. It was mainly hydrophobic forces, disulfide bonding, and hydrogen bonding for U-DRVPs, whereas it was mainly hydrogen bonding, hydrophobic forces, and ionic bonding for C-DRVPs.

### 3.5. Functional Properties of DRVPs

#### 3.5.1. Solubility

**Figure 5:**
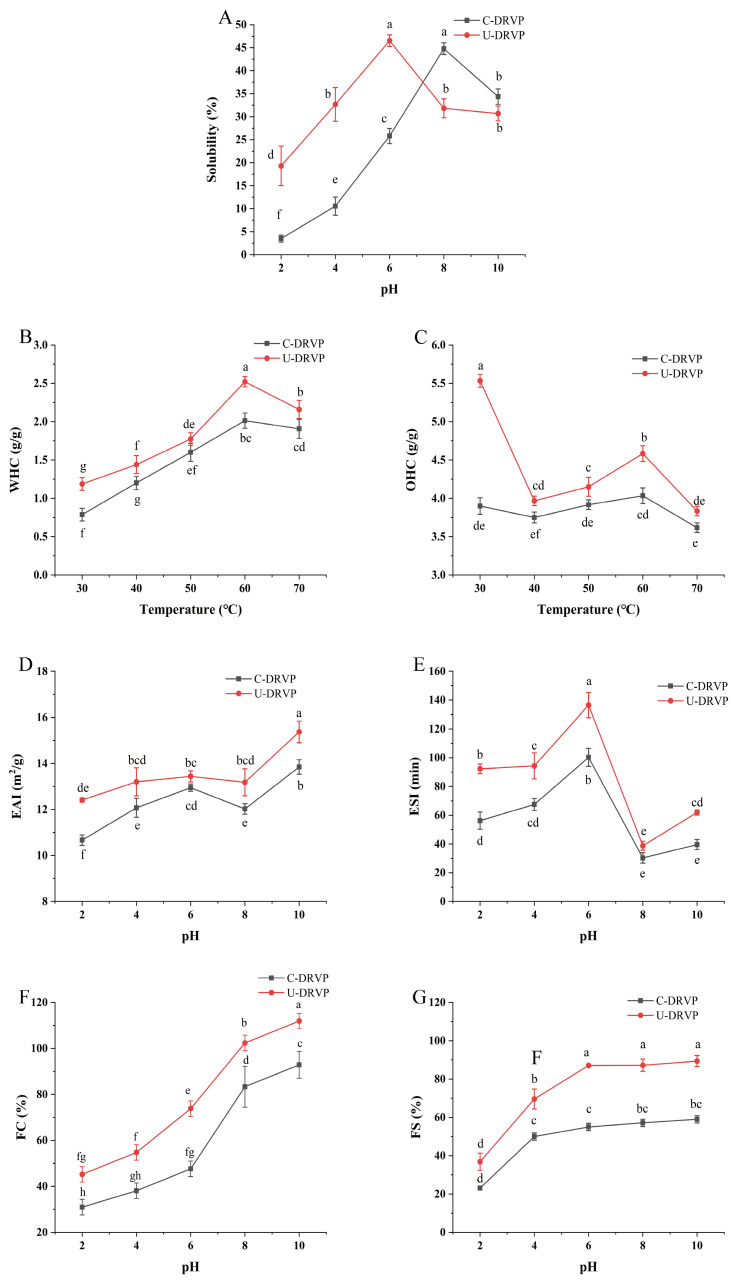
Functional properties of C-DRVP and U-DRVP. (**A**) Solubility, (**B**) WHC, (**C**) OHC, (**D**) EAI, (**E**) ESI, (**F**) FC, and (**G**) FS. Note: Different letters indicate a significant different between the means of any two groups (*p* < 0.05). C-DRVPs, *D. rubrovolvata* volva proteins extracted using conventional alkaline extraction without UAE; U-DRVPs, *D. rubrovolvata* volva proteins extracted using UAE.

Protein solubility is the most crucial functional characteristic, influencing properties like emulsification, foaming, and gelation [21]. These properties determine the practical uses of proteins in various food systems. In this study, solubility was measured at different pH levels (ranging from 2 to 10) to simulate acidic, neutral, and alkaline conditions in food matrices (Figure 5A). Due to their close proximity to the isoelectric point, both proteins were least soluble at pH 2. As the pH deviated from the isoelectric point, the solubility increased dramatically, which was related to the homo-charge repulsion [21]. Interestingly, compared to C-DRVPs, U-DRVPs exhibited better solubility under acidic conditions (pH < 7), while the results were opposite under alkaline conditions. Several findings found that the higher solubility of sonicated proteins was dependent on size reduction [9]. However, the present observations suggested that the relationship between particle size and solubility may not be so straightforward [32]. Through sonocavitation, U-DRVPs partially unfolded, exposing its internal hydrophilic groups. As a result, U-DRVPs were able to interact more strongly with the surrounding water molecules [9].

#### 3.5.2. WHC and OHC

WHC and OHC are very important as they severely affect the taste and texture of diverse food types [33]. As illustrated in Figure 5B, the WHC of DRVP increased with rising temperature regardless of sonication, reaching the maximum value at 60 °C for both U-DRVP (2.52 g/g) and C-DRVP (2.01 g/g). Continuing to increase the temperature, WHC gradually declined. U-DRVP presented higher WHC than C-DRVP throughout the temperature range tested. This may be due to the transient high temperature and pressure created by the cavitation bubble burst, which loosened the spatial structure of U-DRVP, increasing the contact area of water molecules with the hydrophilic groups in U-DRVP, and subsequently improving WHC [33]. Additionally, the increase in temperature further encouraged the stretching of DRVP, which contributed to the further enhancement of WHC. However, excessive temperature severely damaged the protein structures, resulting in a significant reduction in WHC.

Likewise, U-DRVP had higher OHC than C-DRVP. In general, lipids bind to proteins via hydrophobic forces between fatty chains and nonpolar amino acid side chains. Thus, it was hypothesized that U-DRVP with higher hydrophobicity would have an improved lipid-binding capacity [34,35]. OHC of U-DRVP and C-DRVP peaked at the initial temperature (30 °C) at 5.53 g/g and 3.90 g/g (Figure 5C), respectively. Keeping the temperature elevated, OHC showed a trend of decreasing–increasing–decreasing, but always lower than that at the starting temperature. This suggests that high temperature was unfavorable for oil uptake by DRVPs. Changes in physicochemical properties and spatial structure may be responsible for the complex temperature effects on OHC.

#### 3.5.3. Emulsifying Properties

Emulsification characterized by EAI and ESI is an important quality index in the food manufacturing process. EAI refers to the ability of proteins to promote emulsion formation, whilst ESI reflects the proteins property to keep the emulsion stable over time [36]. As displayed in Figure 5D, both pH and UAE had significant effects on the EAI of DRVP. The EAI of both DRVPs improved with pH increasing, and U-DRVPs showed a higher EAI over the entire pH range. It was worth noting that the poorer solubility (Figure 5A) and larger particle size (Figure 2D) of U-DRVPs did not prevent them from exhibiting better EAI in alkaline conditions as compared to C-DRVPs. Thus, in this case, the EAI may be influenced by the combination of several factors, such as solubility, particle size, spatial conformation, hydrophilicity, and lipophilicity [37,38,39]. Under the effect of sonication cavitation and shear force, the spatial structure of U-DRVP was more stretched, exposing more hydrophilic and hydrophobic groups [36]. As a result, the rapid migration and absorption of U-DRVPs at the oil–water interface lowered the interfacial tension and ultimately improved the emulsification [35]. Consistent with the results of EAI, U-DRVPs also had higher ESI values than C-DRVPs over the given pH range, with a maximum value of 136.53 min at pH = 6. In conclusion, U-DRVPs with high EAI and ESI values possess improved emulsification properties. Sauces, dressings, baked foods, and meat analogs can all make use of it as an emulsifier.

#### 3.5.4. Foaming Properties

The foaming properties of proteins are essential for the production of foaming foods such as ice cream, cakes, and beer [22]. Two indicators serve to evaluate the foaming properties of proteins, FC and FS. The FC of both U-DRVPs and C-DRVPs grew with increasing pH. Their maximum values were 111.90% and 92.85% at pH 10, respectively (Figure 5F). UAE was effectively used to improve FC in the assayed pH range. The possible explanation involved a decrease in the proportion of α-helix, the densest secondary structure (Figure 3C). As a result, the flexibility of the proteins increased and the buried hydrophobic groups were uncovered [40]. U-DRVPs were then rapidly dispersed at the air/water interface, entrapping the air bubbles and improving foaming performance [40]. Analogously, UAE substantially increased the FS of DRVPs (Figure 5G). Under alkaline conditions, despite being less soluble than C-DRVPs, U-DRVPs retained a higher FS. A possible explanation for this contradictory result might be that U-DRVPs were more uniform in size compared to C-DRVPs. This contributed to the reduction in intermolecular forces and interfacial surface tension, making it easier to build a firm, thick, cohesive, elastic and continuous film over the dispersed bubbles, thus stabilizing them [22].

## 4. Conclusions

The present study demonstrated that UAE can significantly improve the protein extraction rate and functional properties of DRVPs. Under the optimal process conditions, the extraction rate of DRVPs can reach 43.34%. Meanwhile, the results of SDS-PAGE patterns, FTIR, H_0_, and SH contents indicated that the primary structure of U-DRVPs remained unchanged, whereas the secondary and tertiary structures varied obviously. Turbidity and particle size results suggested that ultrasound promoted the reorganization of U-DRVPs to form aggregates with increased particle size, which were maintained mainly by hydrophobic forces, disulfide bonding, and hydrogen bonding. Moreover, water/oil-holding ability, as well as the emulsifying and foaming properties of DRVPs were significantly enhanced using UAE. It was ascribed to the comprehensive effects induced by ultrasonic cavitation. Specifically, it referred to the changes in the advanced structure, molecular interactions, and physicochemical properties of DRVPs, rather than the reduction in particle size commonly assumed. In conclusion, these findings provided valuable information on *D. rubrovolvata* volva as a potential protein source. Further research should focus on the scale-up of DRVP manufacturing and the development of corresponding ultrasound equipment for its future application in the food industry.

## Figures and Tables

**Table 1:** Amino acid compositions of C-DRVPs and U-DRVPs.

Amino Acids	Absolute Quantities (mg/g)		Relative Quantities (%)	
	C-DRVPs	U-DRVPs	C-DRVPs	U-DRVPs
Histidine (His)	16.98 ^b^	18.79 ^a^	2.48 ^A^	2.50 ^A^
Isoleucine (Ile)	39.04 ^b^	43.01 ^a^	5.71 ^A^	5.72 ^A^
Leucine (Leu)	53.96 ^b^	60.54 ^a^	7.89 ^B^	8.05 ^A^
Lysine (Lys)	24.16 ^b^	27.65 ^a^	3.53 ^B^	3.68 ^A^
Methionine (Met)	9.06 ^b^	9.89 ^a^	1.33 ^A^	1.31 ^A^
Phenylalanine (Phe)	47.28 ^b^	51.68 ^a^	6.91 ^A^	6.87 ^A^
Threonine (Thr)	52.64 ^b^	56.99 ^a^	7.70 ^A^	7.58 ^B^
Valine (Val)	43.74 ^b^	47.98 ^a^	6.39 ^A^	6.38 ^A^
Aspartic acid (Asp)	85.52 ^b^	93.15 ^a^	12.51 ^A^	12.39 ^B^
Glutamic acid (Glu)	77.70 ^b^	86.48 ^a^	11.36 ^A^	11.50 ^A^
Serine (Ser)	46.66 ^b^	50.81 ^a^	6.82 ^A^	6.76 ^B^
Cystine (Cys)	2.26 ^b^	2.44 ^a^	0.33 ^A^	0.33 ^A^
Glycine (Gly)	40.54 ^b^	44.51 ^a^	5.93 ^A^	5.92 ^A^
Tyrosine (Tyr)	27.02 ^b^	29.76 ^a^	3.95 ^A^	3.96 ^A^
Arginine (Arg)	26.24 ^b^	29.42 ^a^	3.84 ^B^	3.91 ^A^
Alanine (Ala)	44.86 ^b^	49.66 ^a^	6.56 ^A^	6.60 ^A^
Proline (Pro)	46.24 ^b^	49.15 ^a^	6.76 ^A^	6.54 ^B^
HAA	324.72 ^b^	356.41 ^a^	49.25 ^A^	49.05 ^B^
EAA	286.86 ^b^	316.53 ^a^	41.94 ^B^	42.09 ^A^
NCAA	163.22 ^b^	179.63 ^a^	23.87 ^A^	23.89 ^A^
PCAA	67.38 ^b^	75.85 ^a^	9.85 ^B^	10.09 ^A^
Total	683.90 ^b^	751.89 ^a^	100.00 ^A^	100.00 ^A^

Note: HAA, hydrophobic amino acids (Thr, Ala, Val, Met, Leu, Ile, Phe, Pro); EAA, essential amino acids (Thr, Val, Met, Ile, Leu, Phe, Lys, His); NCAA, negatively charged amino acids (Asp, Glu); and PCAA, positively charged amino acids (Arg, His, Lys). Means with different superscript lowercase (uppercase) letters within the same line are significantly different (*p* < 0.05). C-DRVP, *D. rubrovolvata* volva proteins extracted using conventional alkaline extraction without UAE; U-DRVPs, *D. rubrovolvata* volva proteins extracted using UAE.

## Data Availability

The original contributions presented in the study are included in the article/Supplementary Material, further inquiries can be directed to the corresponding authors.

## Supplementary Materials

The following supporting information can be downloaded at: https://www.mdpi.com/article/10.3390/foods13081265/s1, Figure S1: Effects of different dissociation agents on particle size distribution of U-DRVP; Figure S2: Effects of different dissociation agents on particle size distribution of C-DRVP; Table S1: The factors and levels of the orthogonal test; Table S2: Orthogonal array design with experimental results; Table S3: Variance analysis of orthogonal test.
