# Supplementary material for: Ultrasonic-Assisted Extraction of Dictyophora rubrovolvata Volva Proteins: Process Optimization, Structural Characterization, Intermolecular Forces, and Functional Properties
Source: Foods. 2024 Apr 20;13(8):1265. doi: 10.3390/foods13081265 (PMC11049406; doi:10.3390/foods13081265)
Supplement: Supplementary file 1 [file foods-13-01265-s001.zip › foods-2944270-supplementary.pdf]

# Supporting Information

**Ultrasonic Assisted Extraction of *Dictyophora rubrovolvata* Volva Proteins: Process Optimization, Structural Characterization, Intermolecular Forces, and Functional Properties**

Yongqing Zhang[a], Shinan Wei[b], Qinqin Xiong[b], Lingshuai Meng[a], Ying Li[a], Yonghui Ge[a], Ming Guo[c], Heng Luo[b,**], Dong Lin[a,*]

[a]Guizhou Higher Education Key Laboratory of Functional Food, Guizhou Engineering Research Center for Fruit Processing, College of Food Science and Engineering, Guiyang University, Guiyang 550005, China

[b]State Key Laboratory of Functions and Applications of Medicinal Plants, Guizhou Medical University, Guiyang 550014, China.

[c]Guizhou Jin Chan Da Shan Biotechnology Company Limited, Nayong 553300, China

*Corresponding author. College of Food Science and Engineering, Guiyang University, Guiyang 550005, China.

** Corresponding author. State Key Laboratory of Functions and Applications of Medicinal Plants, Guizhou Medical University, Guiyang 550014, China.

E-mail addresses: gyulindong@gyu.edu.cn (D. Lin), luo_heng@gmc.edu.cn (H. Luo).

**Table S1.** The factors and levels of the orthogonal test

| Factors | Levels | | |
|---|---|---|---|
| | 1 | 2 | 3 |
| A. pH | 10 | 10.5 | 11 |
| B. Time (min) | 20 | 25 | 30 |
| C. Ultrasonic power (W) | 440 | 495 | 550 |
| D. Material to solvent (g/mL) | 1:15 | 1:20 | 1:25 |

**Table S2.** Orthogonal array design with experimental results

| No. | A | B | C | D | Proteins extraction rate (%) |
|---|---|---|---|---|---|
| 1 | 10 | 20 | 440 | 1:15 | 30.81±0.93 |
| 2 | 10 | 25 | 495 | 1:20 | 33.84±1.05 |
| 3 | 10 | 30 | 550 | 1:25 | 35.93±1.49 |
| 4 | 10.5 | 20 | 495 | 1:25 | 35.75±0.96 |
| 5 | 10.5 | 25 | 550 | 1:15 | 38.11±1.69 |
| 6 | 10.5 | 30 | 440 | 1:20 | 34.09±0.52 |
| 7 | 11 | 20 | 550 | 1:20 | 42.84±0.65 |
| 8 | 11 | 25 | 440 | 1:25 | 38.62±1.01 |
| 9 | 11 | 30 | 495 | 1:15 | 39.85±1.40 |
| $k_1$ | 33.53 | 36.47 | 34.51 | 36.26 | |
| $k_2$ | 35.98 | 36.86 | 36.48 | 36.92 | |
| $k_3$ | 40.44 | 36.62 | 38.96 | 36.77 | |
| R | 6.91 | 0.39 | 4.45 | 0.66 | |

**Table S3.** Variance analysis of orthogonal test.

| Factors | Sum of square | Degree of freedom | Mean of square | *F*-value | *P*-value | Significant |
|---|---|---|---|---|---|---|
| A | 211.02 | 2 | 110.51 | 57.03 | 0.0001 | ** |
| B | 0.71 | 2 | 0.36 | 0.18 | 0.83 | |
| C | 89.62 | 2 | 44.81 | 23.12 | 0.0001 | ** |
| D | 2.18 | 2 | 1.09 | 0.56 | 0.58 | |
| Error | 34.88 | 18 | 1.94 | | | |
| Total | 36613.95 | 27 | | | | |

** means that the differences are extremely significant, $P < 0.01$.

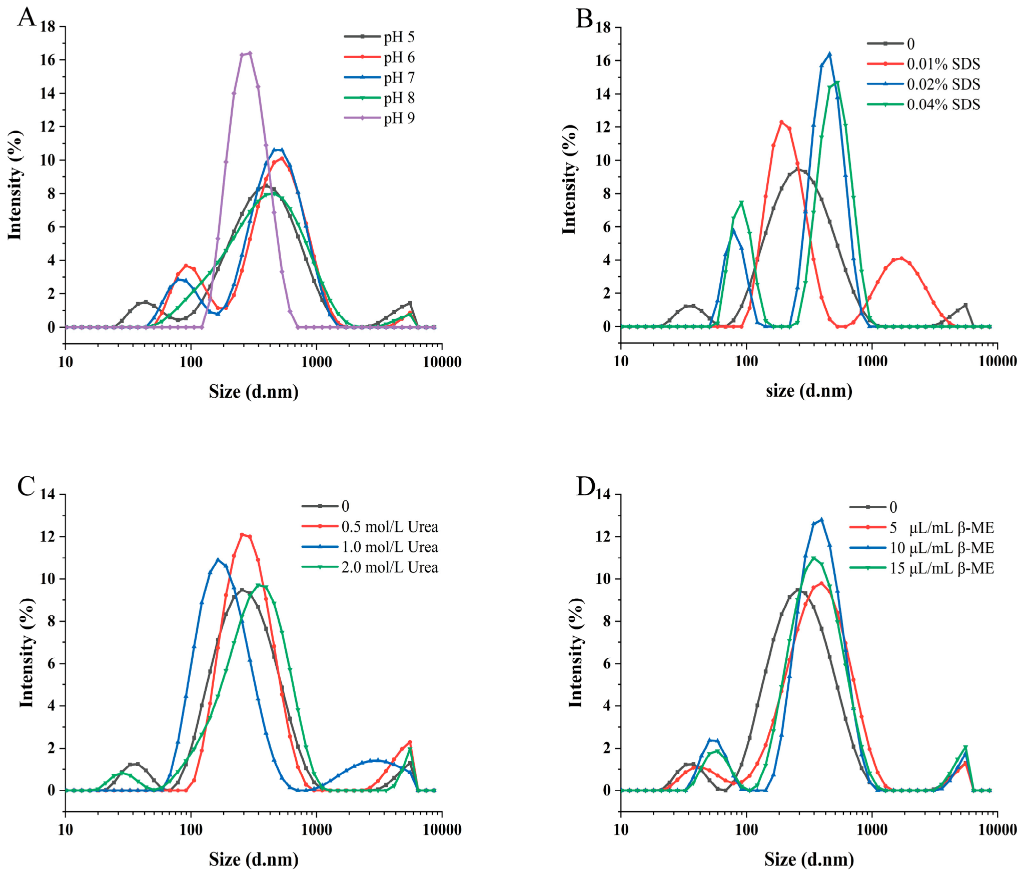


**Figure S1.** Effects of different dissociation agents on particle size distribution of U-DRVP. (A) pH, (B) SDS, (C) urea, and (D) β-ME. Note: U-DRVP, *D. rubrovolvata* volva proteins extracted by UAE.

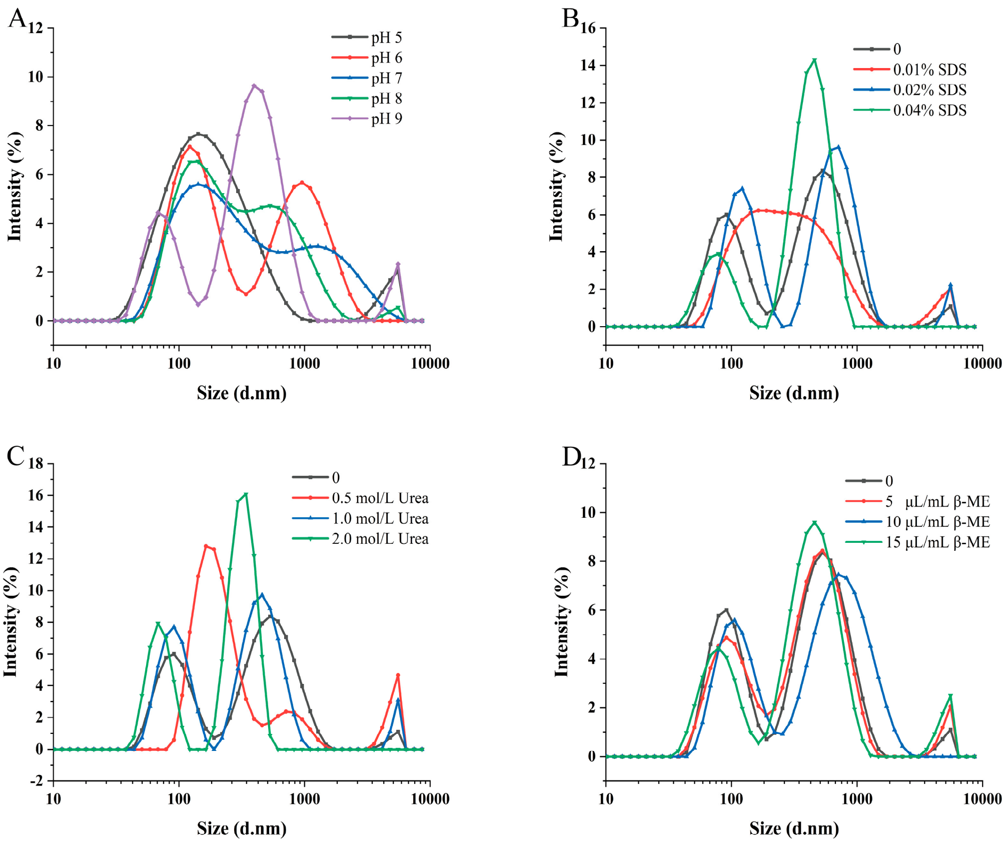


**Figure S2.** Effects of different dissociation agents on particle size distribution of C-DRVP. (A) pH, (B) SDS, (C) urea, and (D) β-ME. Note: C-DRVP, *D. rubrovolvata* volva proteins extracted by conventional alkaline extraction without UAE.
